# Clinical exploration of first-line therapy in metastatic lung adenocarcinoma patients with negative or low PD-L1 expression: a retrospective cohort study

**DOI:** 10.3389/fimmu.2026.1836760

**Published:** 2026-06-03

**Authors:** Kun Xu, Ruoxue Cai, Zijian Sun, Cenzhu Wang, Jun Bao

**Affiliations:** 1 Department of Oncology, Jiangsu Institute of Cancer Research, The Affiliated Cancer Hospital of Nanjing Medical University, Jiangsu Cancer Hospital, Nanjing, China; 2 Department of Oncology, National Cancer Center/National Clinical Research Center for Cancer/Cancer Hospital, Chinese Academy of Medical Sciences and Peking Union Medical College, Beijing, China; 3 Department of Oncology, The Affiliated Wuxi People’s Hospital of Nanjing Medical University, Wuxi People’s Hospital, Wuxi Medical Center, Nanjing Medical University, Wuxi, China

**Keywords:** anti-angiogenic agents, driver-gene-negative, metastatic lung adenocarcinoma, PD-1/PD-L1 inhibitors, PD-L1 expression

## Abstract

**Background:**

Either Programmed cell death protein 1 (PD-1)/Programmed cell death-Ligand protein 1 (PD-L1) inhibitors plus chemotherapy or anti-angiogenic agents plus chemotherapy has become first-line therapy in negative or low PD-L1 patients with driver-gene-negative metastatic lung adenocarcinoma. PD-1/PD-L1 inhibitors plus anti-angiogenic agents with chemotherapy is also an option. However, there are no large studies evaluating the advantages and disadvantages of the three treatment strategies.

**Methods:**

Totally 163 stage IV lung adenocarcinoma patients with negative or low PD-L1 expression who received first-line treatment at Nanjing Medical University Affiliated Cancer Hospital from November 1, 2019 to December 1, 2023 were analyzed. We evaluated the difference in efficacy of multiple treatment regimens.

**Results:**

57 patients received anti-angiogenic agents plus chemotherapy (AC group), 69 patients received PD-1/PD-L1 inhibitors plus chemotherapy (PC group) and 37 patients received PD-1/PD-L1 inhibitors plus anti-angiogenic agents with chemotherapy (APC group). The median progression-free survival (mPFS) was 6.3 months, 8.7 months and 13.8 months in AC, PC and APC group, respectively. The APC group significantly prolonged patients’ PFS compared to the AC and PC groups. APC group and PC group had no significant difference in overall survival (OS) compared to AC group. Subgroup analysis showed that APC group significantly lowered the risk of disease progression compared to AC group in the PD-L1-negative subgroup. The incidence of grade 3–4 adverse events (AEs) was similar in three groups.

**Conclusions:**

Among the first-line treatment regimens for metastatic lung adenocarcinoma patients with negative or low PD-L1 expression, PD-1/PD-L1 inhibitors plus anti-angiogenic agents with chemotherapy showed significant benefit in PFS compared to anti-angiogenic agents plus chemotherapy and PD-1/PD-L1 inhibitors plus chemotherapy.

## 1 Introduction

Lung adenocarcinoma is the most common histological type of non-small cell lung cancer, accounting for about 40% (1). Targeted therapy has become the first-line treatment option for positive driver gene (epidermal growth factor receptor (EGFR) or anaplastic lymphoma kinase (ALK)) patients with advanced lung adenocarcinoma (2, 3). Previously, chemotherapy has been the standard treatment protocol in patients with driver-gene-negative advanced lung adenocarcinoma. With the clinical use of PD-1/PD-L1 inhibitors, the treatment of advanced lung adenocarcinoma patients with driver-gene-negative has seen a new dawn. Metastatic lung adenocarcinoma patients with negative driver-genes always suffered from rapid progression and poor prognosis. Therefore, the choices of first-line therapy in metastatic lung adenocarcinoma patients deserve for more attention and exploration.

The KEYNOTE-024 trial demonstrated improved OS and PFS for pembrolizumab as first-line therapy compared to chemotherapy alone in metastatic NSCLC with PD-L1 ≥ 50% (4, 5). Subgroup analysis showed that the primary beneficiary population remained those with PD-L1>50% though the KEYNOTE-042 trial expanded enrollment (6). Pembrolizumab plus chemotherapy exhibited survival benefits regardless of PD-L1 expression status in the KEYNOTE-407 study (7). The beneficiary population of pembrolizumab was expanded from patients with high PD-L1 levels to all expressions of PD-L1. Although pembrolizumab in combination with chemotherapy is indicated for patients with negative or low PD-L1 expression, and patients with PD-L1-low NSCLC also have the possibility to benefit from immunotherapy, the main beneficiaries of subgroup analysis are still patients with high PD-L1 expression. The IMPOWER 110 study reported significantly longer OS for atelelizumab than platinum-based chemotherapy in NSCLC with high PD-L1 expression (8). The IMPOWER 150 trial is the first study to compare the efficacy of two regimens, PD-1/PD-L1 inhibitors plus chemotherapy and anti-angiogenic agents plus chemotherapy, in patients with low PD-L1 expression, but OS showed no statistically significant difference between the two groups in subgroup analysis (9).

The ECOG 4599 and BEYOND study showed that bevacizumab in combination with chemotherapy significantly improved OS compared with chemotherapy alone in patients with driver-gene-negative advanced non-squamous NSCLC. However, because the PD-L1 expression status of most patients was unknown, whether bevacizumab combined with chemotherapy has clinical benefit in patients with low PD-L1 expression needs to be investigated (10, 11).

Currently, lung adenocarcinoma patients with high PD-L1 expression and negative driver genes benefit significantly from PD-1/PD-L1 inhibitors, but the effect is inexact in patients with low and negative PD-L1 expression. Current optional clinical treatments include PD-1/PD-L1 inhibitors plus chemotherapy, anti-angiogenic agents plus chemotherapy and etc., but there is no definitive conclusion about the standard treatment regimen for this population. And no head-to-head comparative trials have directly evaluated the efficacy of these treatment options in the population. Therefore, we conducted this retrospective study to optimize the treatment regimen for patients with low or negative PD-L1 expression and driver-gene-negative metastatic lung adenocarcinoma, by comparing the efficacy of PD-1/PD-L1 inhibitors and anti-angiogenic agents. Meanwhile, we evaluated the efficacy and safety of PD-1/PD-L1 inhibitors plus anti-angiogenic agents with chemotherapy.

## 2 Materials and methods

### 2.1 Study design and patients

**Figure 1:**
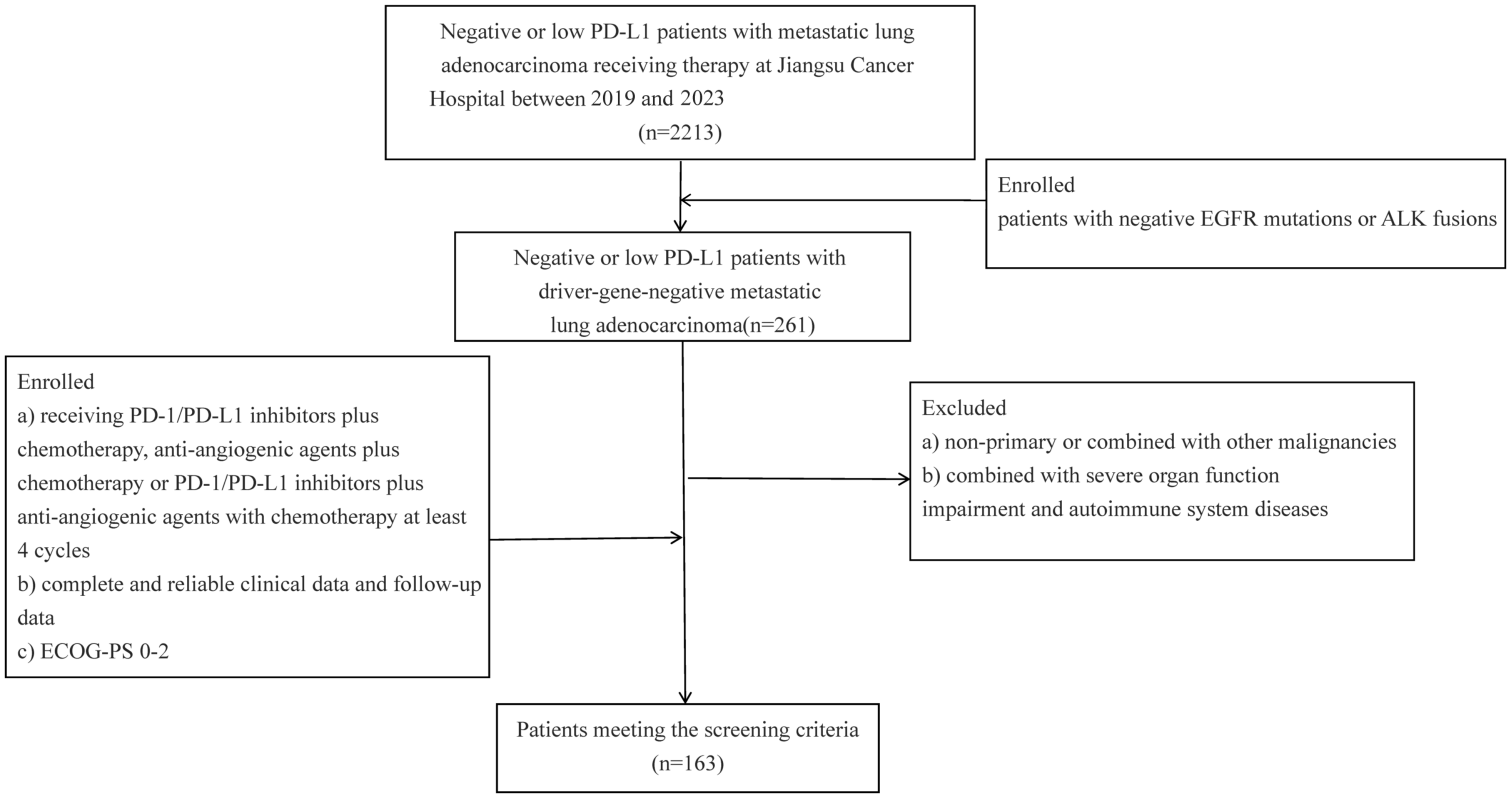
Flowchart.

The data of patients with metastatic lung adenocarcinoma receiving first-line therapy at Nanjing Medical University Affiliated Cancer Hospital from November 1, 2019 to December 1, 2023 were retrospectively reviewed in this study. The eligibility standards were as follows: (1) pathologically confirmed primary lung adenocarcinoma; (2) receiving PD-1/PD-L1 inhibitors plus chemotherapy, anti-angiogenic agents plus chemotherapy or PD-1/PD-L1 inhibitors plus anti-angiogenic agents with chemotherapy at least 4 cycles; (3) PD-L1 TPS<50%; (4) clinical stage IV (the 8th edition of the AJCC TNM staging system); (5) complete and reliable clinical data and follow-up data. The exclusion standards were as follows: (1) patients with EGFR mutations or ALK fusions; (2) combined with severe organ function impairment and autoimmune system diseases. Immune checkpoint inhibitors included PD-1 inhibitors (pembrolizumab, toripalimab, tislelizumab, sintilimab, camrelizumab) and PD-L1 inhibitors (atezolizumab). PD-L1 expression was defined as the percentage of tumor cells with membranous PD-L1-positive staining (TPS) using 22C3 antibody by immunohistochemistry (IHC). The anti-angiogenic drug is bevacizumab. All included patients were treated with chemotherapy with a platinum-containing regime, including platinum analogs (carboplatin, cisplatin, nedaplatin), pemetrexed and paclitaxel analogs (docetaxel, paclitaxel, albumin paclitaxel, paclitaxel liposomes) (**Figure 1**).

This study received approval from the Ethics Committee of Jiangsu Cancer Hospital (approval number 2023-033). The local ethics committee waived written patient consent due to the retrospective design. Verbal consent was obtained from patients or their proxies before starting the phone interview. We ensured confidentiality when extracting and analyzing patients’ data. All procedures of this study were in accordance with the Declaration of Helsinki (as revised in 2013).

### 2.2 Data collection and assessment

Data were collected from medical records. Patients who had not visited the hospital for three months were followed up by telephone contact to get information including tumor recurrence and survival. We collected general and clinical information (tumor site, PD-L1 expression, metastatic site and treatment plan). Response Evaluation Criteria in Solid Tumors (RECIST) version 1.1 was applied to classify effectiveness into four categories: complete response (CR), partial response (PR), stable disease (SD) and disease progression (PD). Objective response rate (ORR) indicates CR and PR. Disease control rate (DCR) indicates CR, PR and SD.

The primary endpoints of this study were OS and PFS and secondary endpoints were DCR and ORR, assessed by the investigators for disease response. Adverse events were evaluated by the National Cancer Institute Common Terminology Criteria for Adverse Events (CTCAE) version 5.0. PFS was defined as the time from the patient’s first treatment to disease progression or last follow-up. OS was defined as the time from the patient’s first treatment to death or last follow-up.

**Figure 2:**
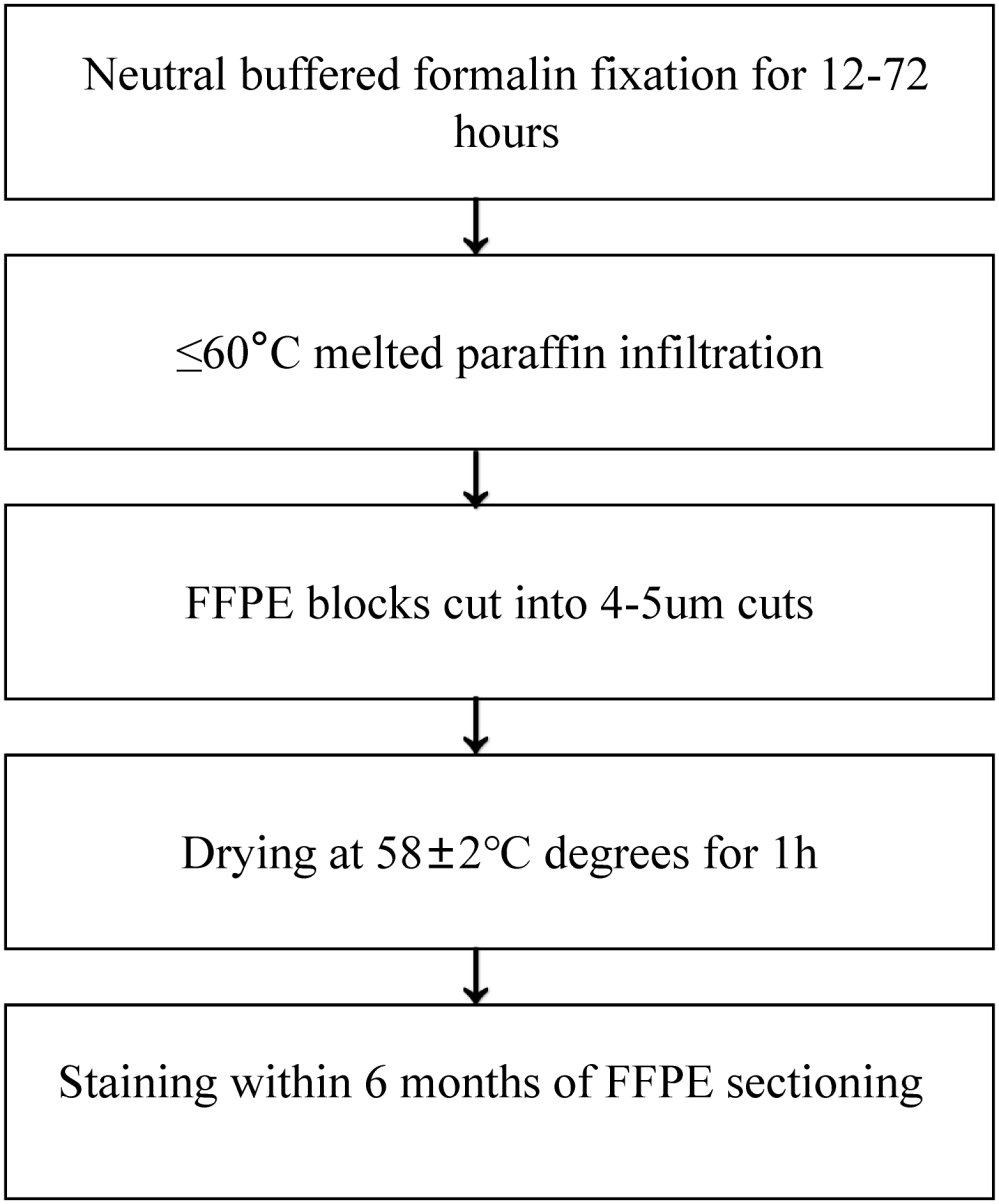
PD-L1 detection process.

Tumor samples were obtained by tissue biopsy at the time of disease confirmation. PD-L1 expression was detected using 22C3 antibody by IHC and classified as PD-L1 TPS <1%, 1%-49% and ≥50% according to the test results. We defined PD-L1 TPS <1% as negative and PD-L1 TPS 1%-49% as low expression based on the results of current large studies (**Figure 2**).

### 2.3 Statistical analysis

All statistical analyses were performed in SPSS (version 26.0), GraphPad Prism ((V.7.0; GraphPad Software) and R environment v.4.1.3. Continuous and categorical variables were described by median (interquartile range) and frequency (percentage) respectively. We used the Kaplan-Meier method to plot survival curves and the log-rank test to compare differences. Differences between groups were tested by χ 2 or Fisher’s exact test (for categorical variables). Cox regression was used for both univariate and multivariate analyses. We used subgroup analyses to assess the association between groups and the risk of disease progression and death in different subgroups. Two-sided P values < 0.05 were considered statistically significant.

## 3 Results

### 3.1 Patient characteristics

A total of 163 patients were analyzed, with a mean age of 63.3 ± 9.5 years. The female patients accounted for 24.5% while the male patients accounted for 75.5%. 57 (35.0%) patients received anti-angiogenic agents plus chemotherapy (AC group), 69 (42.3%) patients received PD-1/PD-L1 inhibitors plus chemotherapy (PC group) and 37 (22.7%) patients received PD-1/PD-L1 inhibitors plus anti-angiogenic agents with chemotherapy (APC group).

All patients had PD-L1 TPS <50%, with 64 (39.3%) PD-L1 TPS <1% and 99 (60.7%) PD-L1 TPS of 1-49%. Overall, the clinical characteristics of the three groups of patients were comparable. All patients had adenocarcinoma histology and were negative for EGFR and ALK gene status by receiving next-generation sequencing panels. Baseline characteristics of patients in the three groups included gender, age, ECOG score, smoking history, metastatic site and tumor site (**Table 1**). The differences in all characteristics were not statistically significant.

**Table 1:** Baseline clinical characteristics of patients.

Characteristic	Total (n=163)	AC (n=57)	PC (n=69)	APC (n=37)	p value
Gender					0.192
Male	123 (75.5%)	40 (70.2%)	57 (82.6%)	26 (70.3%)	
Female	40 (24.5%)	17 (29.8%)	12 (17.4%)	11 (29.7%)	
Age	63.3 ±9.5				0.974
<65	81 (49.7%)	28 (49.1%)	35 (50.7%)	18 (48.6%)	
≥65	82 (50.3%)	29 (50.9%)	34 (49.3%)	19 (51.4%)	
ECOG PS					0.397
0-1	143 (87.7%)	49 (86.0%)	59 (85.5%)	35 (94.6%)	
2	20 (12.3%)	8 (14.0%)	10 (14.5%)	2 (5.4%)	
Smoking history					0.866
Yes	61 (37.4%)	20 (35.1%)	26 (37.7%)	15 (40.5%)	
No	102 (62.6%)	37 (64.9%)	43 (62.3%)	22 (59.5%)	
Brain Metastasis					0.366
No	124 (76.1%)	44 (77.2%)	55 (79.7%)	25 (67.6%)	
Yes	39 (23.9%)	13 (22.8%)	14 (20.3%)	12 (32.4%)	
Liver Metastasis					0.617
No	152 (93.3%)	52 (91.2%)	64 (92.8%)	36 (97.3%)	
Yes	11 (6.7%)	5 (8.8%)	5 (7.2%)	1 (2.7%)	
Bone Metastasis					0.560
No	108 (66.3%)	40 (70.2%)	46 (66.7%)	22 (59.5%)	
Yes	55 (33.7%)	17 (29.8%)	23 (33.3%)	15 (40.5%)	
Lung Metastasis					0.163
No	91 (55.8%)	36 (63.2%)	39 (56.5%)	16 (43.2%)	
Yes	72 (44.2%)	21 (36.8%)	30 (43.5%)	21 (56.8%)	
Adrenal Metastasis					0.382
No	144 (88.3%)	53 (93.0%)	59 (85.5%)	32 (86.5%)	
Yes	19 (11.7%)	4 (7.0%)	10 (14.5%)	5 (13.5%)	
Pleura Metastasis					0.054
No	127 (77.9%)	39 (68.4%)	55 (79.7%)	33 (89.2%)	
Yes	36 (22.1%)	18 (31.6%)	14 (20.3%)	4 (10.8%)	
Tumor site					0.602
left	71 (43.6%)	22 (38.6%)	31 (44.9%)	18 (48.6%)	
right	92 (56.4%)	35 (61.4%)	38 (55.1%)	19 (51.4%)	
Radiotherapy history					0.667
No	87 (53.4%)	32 (56.1%)	34 (49.3%)	21 (56.8%)	
Yes	76 (46.6%)	25 (43.9%)	35 (50.7%)	16 (43.2%)	
Radiotherapy site					0.129
Lung	35 (21.5%)	12 (21.1%)	18 (26.1%)	5 (13.5%)	
Bone	21 (12.9%)	5 (8.8%)	7 (10.1%)	9 (24.3%)	
Brain	16 (9.8%)	5 (8.8%)	9 (13.0%)	2 (5.4%)	
Lymphatic node	4 (2.5%)	3 (5.3%)	1 (1.4%)	0 (0.0%)	
PD-L1 TPS					0.143
<1%	64 (39.3%)	27 (47.4%)	27 (39.1%)	10 (27.0%)	
1%-49%	99 (60.7%)	30 (52.6%)	42 (60.9%)	27 (73.0%)	

### 3.2 Cox proportional hazard regression analysis of clinical indicators to predict risk of disease progression and death

In order to explore the relevant factors affecting patients’ prognosis, further relevant studies were conducted. In univariate analysis, PFS was associated with liver metastasis (P = 0.027), smoking history (P<0.001), ECOG score (P<0.001) and bone metastasis (P = 0.003), and OS with gender (P = 0.026), ECOG score (P = 0.004), bone metastasis (P = 0.027) and adrenal metastasis. Both PFS and OS were not significantly related to age, brain metastasis, lung metastasis, pleural metastasis, tumor site and PD-L1 expression status. We then included the factors with P<0.05 in the multifactorial cox analysis, and we found that ECOG score (95%CI: 2.26~4.97, P<0.001), smoking history (95%CI: 1.24~2.76, P = 0.003), and liver metastasis (95%CI: 1.46~5.48, P = 0.002) were independent factors that influenced PFS, and gender (95% CI: 0.23~0.88, P = 0.019), ECOG score (95%CI: 1.26~3.46, P*=*0.004) and bone metastasis (95%CI: 1.11~2.96, P = 0.017) were independent prognostic factors influencing OS (**Tables 2**, **3**; **Supplementary Figures 1**, **S2**).

**Table 2:** Cox proportional hazard regression analysis of clinical indicators to predict risk of disease progression.

Variable	Type	Cox univariate analysis		Cox multivariate analysis	
		HR (95%*CI*)	*P*	HR (95%*CI*)	*P*
Gender	Female or Male	0.96 (0.64~1.45)	0.852		
Age	≥65 or <65	0.94 (0.67~1.33)	0.735		
ECOG	2 or 0~1	2.99 (2.05~4.35)	<0.001*	3.35 (2.26~4.97)	<0.001*
Smoking history	Yes or No	1.91 (1.31~2.78)	<0.001*	1.85 (1.24~2.76)	0.003*
Brain Metastasis	Yes or No	1.43 (0.94~2.18)	0.091		
Liver Metastasis	Yes or No	1.98 (1.07~3.69)	0.027*	2.83 (1.46~5.48)	0.002*
Bone Metastasis	Yes or No	1.72 (1.19~2.47)	0.003*	1.40 (0.95~2.06)	0.090
Lung Metastasis	Yes or No	1.07 (0.75~1.51)	0.722		
Adrenal Metastasis	Yes or No	1.51 (0.89~2.55)	0.126		
Pleura Metastasis	Yes or No	1.38 (0.91~2.08)	0.125		
Tumor site	Right or Left	1.36 (0.96~1.92)	0.083		
PD-L1 TPS	1%~49% or <1%	0.92 (0.64~1.32)	0.651		

Symbol * means that variables are significant with p value < 0.05.

**Table 3:** Cox proportional hazard regression analysis of clinical indicators to predict risk of death.

Variable	Type	Cox univariate analysis		Cox multivariate analysis	
		HR (95%*CI*)	*P*	HR (95%*CI*)	*P*
Gender	Female or Male	0.49 (0.26~0.93)	0.026*	0.45 (0.23~0.88)	0.019*
Age	≥65 or <65	1.20 (0.75~1.91)	0.446		
ECOG	2 or 0~1	2.05 (1.24~3.37)	0.004*	2.09 (1.26~3.46)	0.004*
Smoking history	Yes or No	1.25 (0.77~2.04)	0.366		
Brain Metastasis	Yes or No	1.67 (0.91~3.05)	0.092		
Liver Metastasis	Yes or No	1.35 (0.62~2.95)	0.449		
Bone Metastasis	Yes or No	1.70 (1.06~2.73)	0.027*	1.81 (1.11~2.96)	0.017*
Lung Metastasis	Yes or No	1.05 (0.66~1.67)	0.839		
Adrenal Metastasis	Yes or No	2.18 (1.21~3.91)	0.008*	1.68 (0.92~3.06)	0.093
Pleura Metastasis	Yes or No	1.60 (0.96~2.66)	0.069		
Tumor site	Right or Left	1.25 (0.78~2.00)	0.352		
PD-L1 TPS	1%~49% or <1%	0.93 (0.58~1.50)	0.762		

Symbol * means that variables are significant with p value < 0.05.

### 3.3 Survival analysis

**Figure 3:**
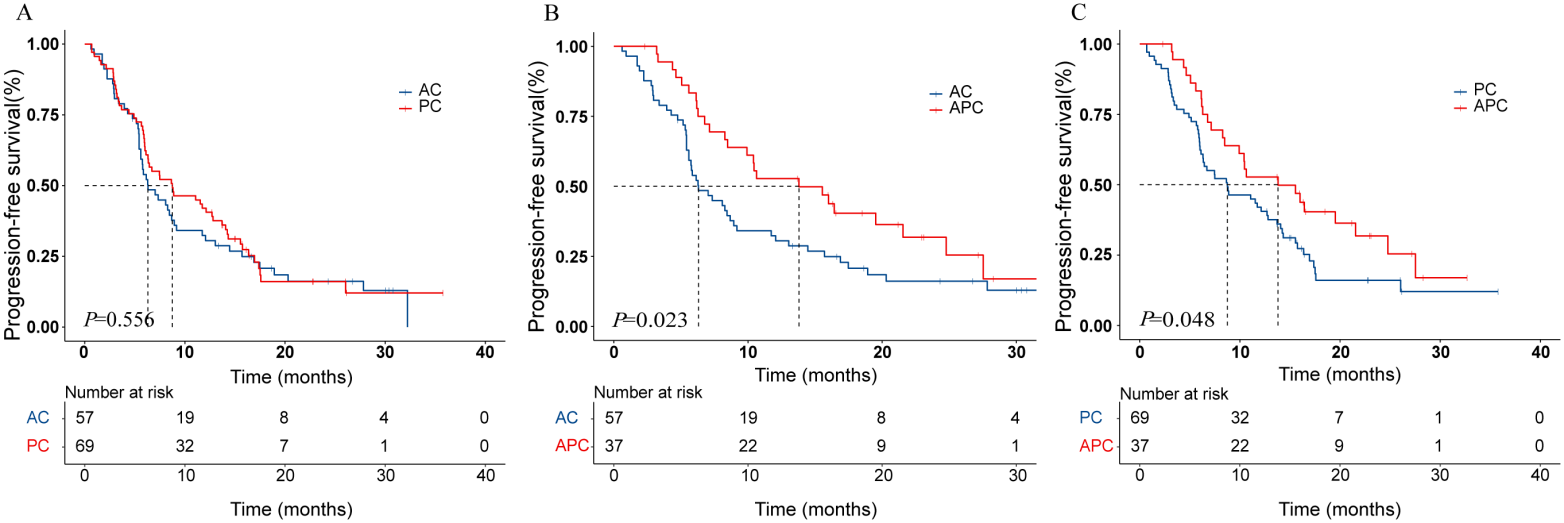
Kaplan-Meier (KM) curves of PFS in the three group.**(A)** AC vs. PC **(B)** APC vs. AC **(C)** APC vs. PC.

**Figure 4:**
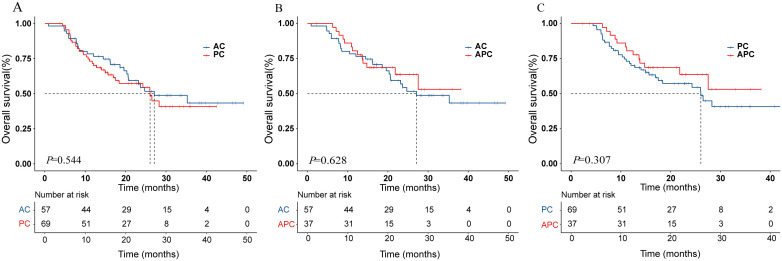
Kaplan-Meier(KM) curves of OS in the three group. **(A)** AC vs. PC **(B)** APC vs. AC **(C)** APC vs. PC

As data cut-off , the median follow-up time of AC, PC and APC group was respectively 30.4 months (27.1~33.6), 26.0 months (22.7~29.4) and 22.3 months (19.6~25.0). Kaplan-Meier analysis showed a median PFS of 6.3 months (95%CI: 4.4~8.2) for patients in AC group, 8.7 months (95%CI: 3.7~13.8) for patients in PC group and 13.8 months (95%CI: 6.1~21.5) for patients in APC group, and a median OS of 27.1 months (95%CI: 14.1~40.1) for patients in AC group and 26.1 months (16.9~35.2) for patients in PC group. The median OS in the APC group has not yet reached. The results of the study showed that there was no significant difference in the risk of disease progression and the risk of death between the AC and PC groups (**Figure 3**).The APC group significantly prolonged the PFS of the patients in comparison with the AC (P = 0.023) and PC groups (P = 0.048), however, the difference was not statistically significant in terms of OS (**Figure 4**).

### 3.4 Stratified analyze

**Figure 5:**
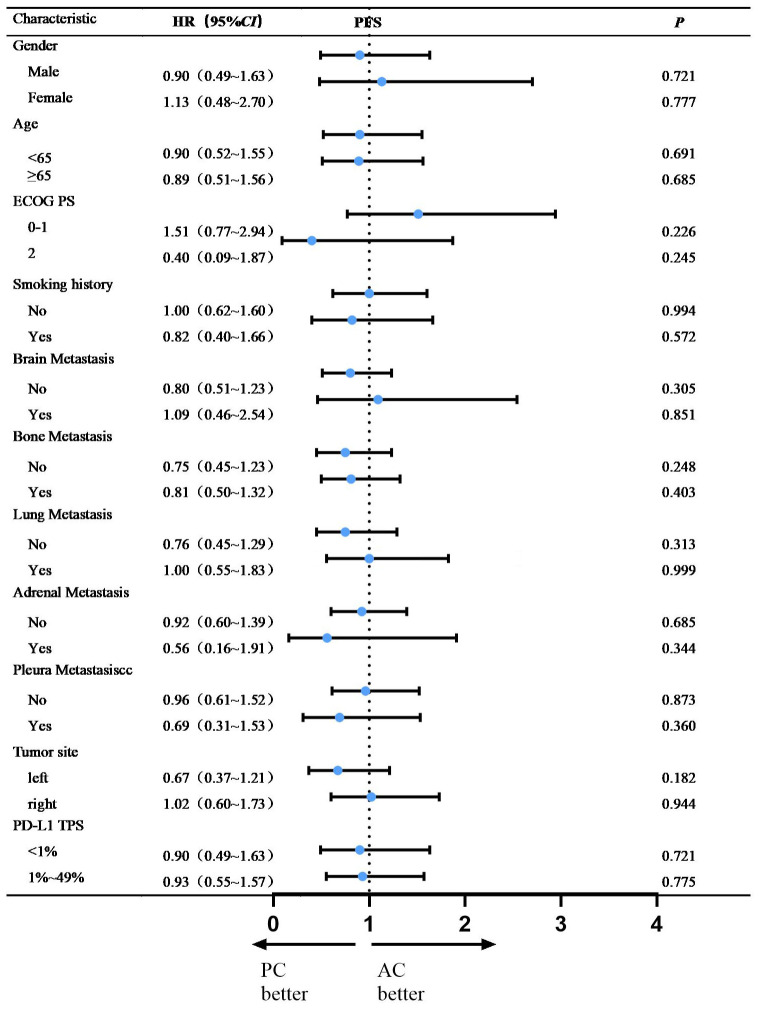
Forest plot of risk of disease progression for patients in PC group compared to AC group in different subgroups.

**Figure 6:**
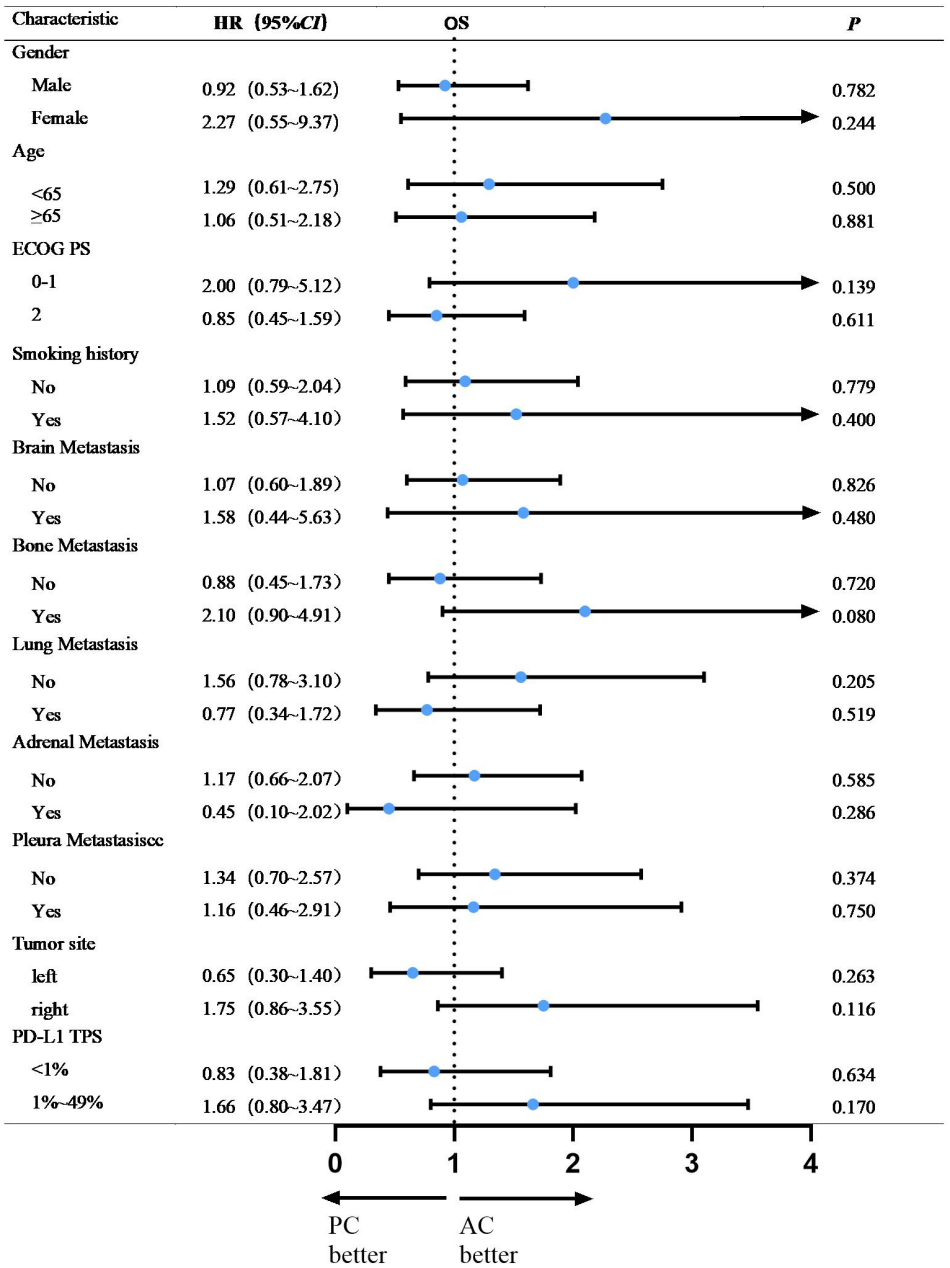
Forest plot of risk of death for patients in PC group compared to AC group in different subgroups.

**Figure 7:**
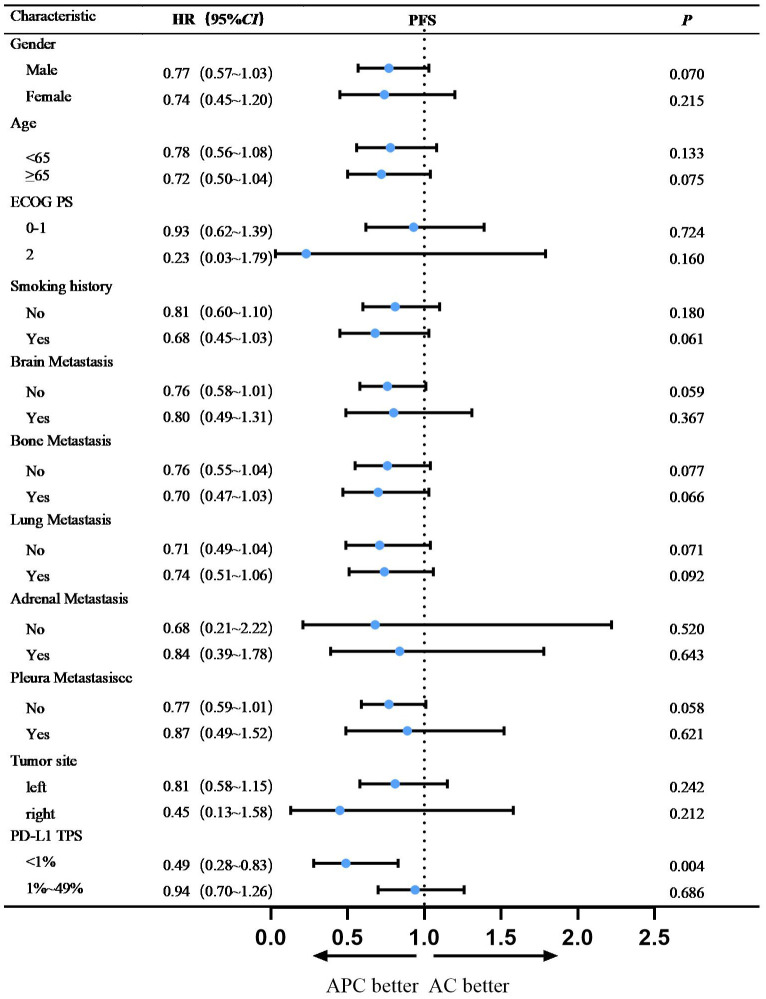
Forest plot of risk of disease progression for patients in AC group compared to APC group in different subgroups.

**Figure 8:**
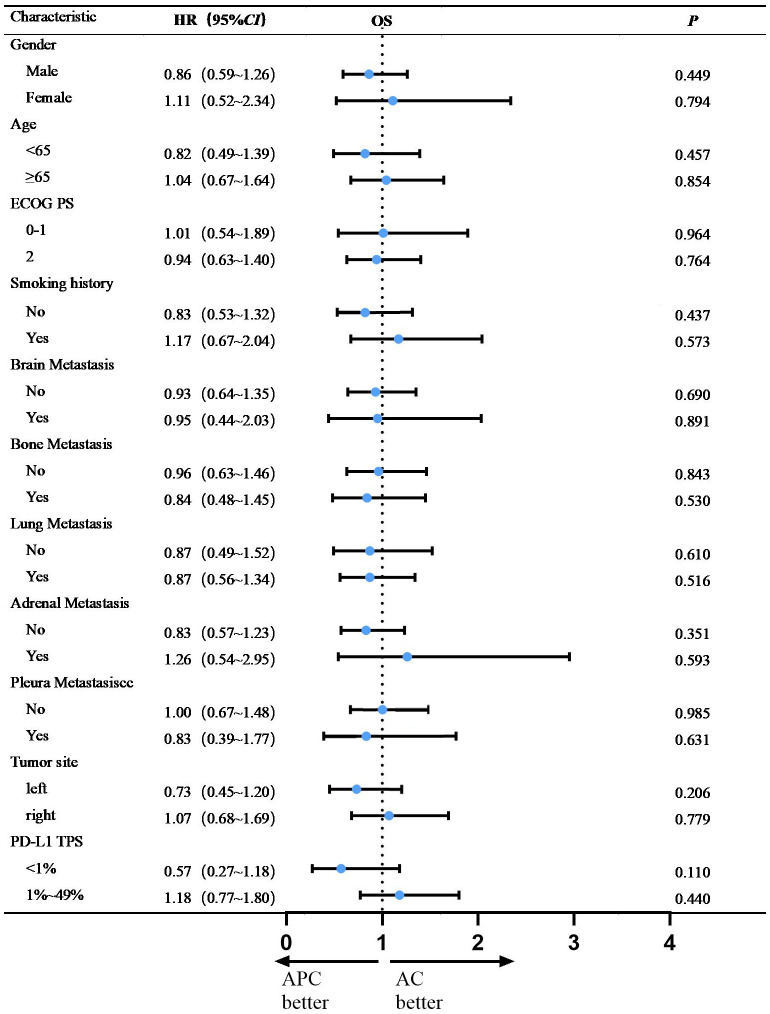
Forest plot of risk of death for patients in APC group compared to AC group in different subgroups.

**Figure 9:**
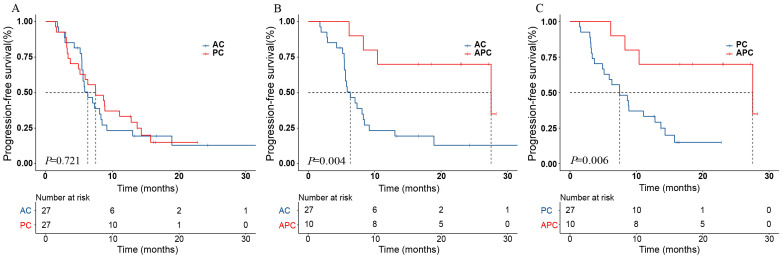
Kaplan-Meier (KM) curves of PFS of patients of PD-L1<1% in PC group versus AC group **(A)**, APC group versus AC group **(B)** and PC group versus APC group **(C)**.

**Figure 10:**
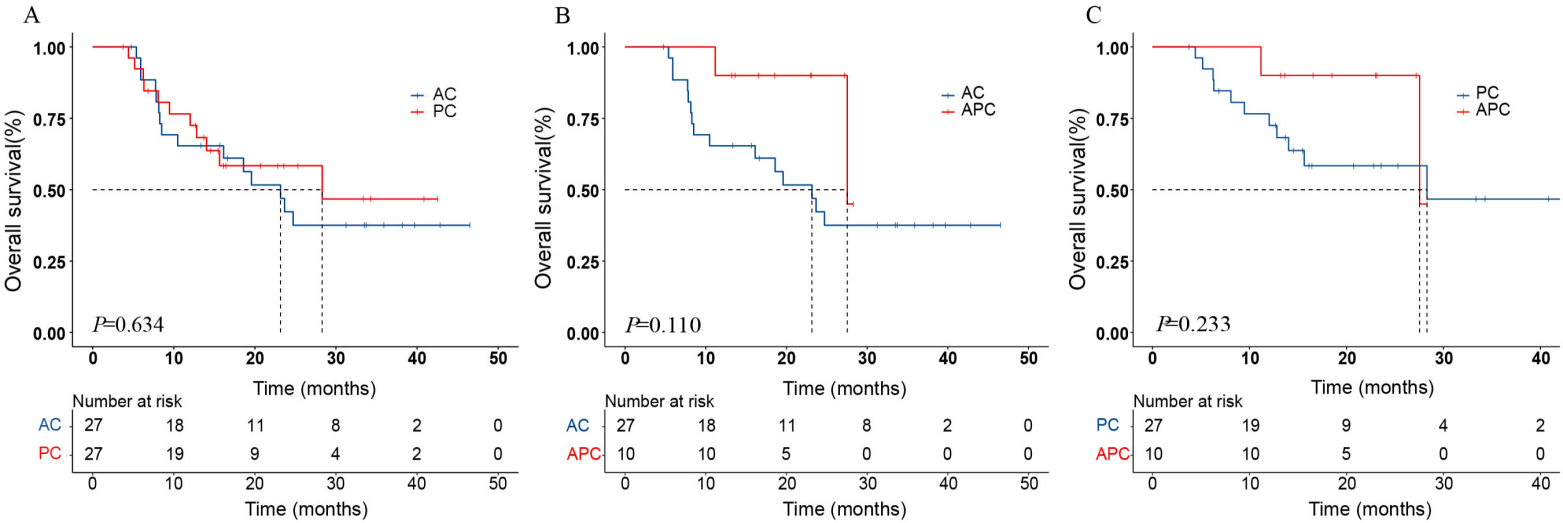
Kaplan-Meier (KM) curves of OS of patients of PD-L1<1% in PC group versus AC group **(A)**, APC group versus AC group **(B)** and PC group versus APC group **(C)**.

We used forest plots to present the risk of disease progression and death for patients in PC and APC group compared to those in AC group in the stratified analyses (**Figures 5**–**8**). In most subgroups, PC and APC group reduced the risk of disease progression relative to the AC group, though not statistically significant. It is worth noting that in the PD-L1-negative subgroup, APC group significantly reduced the risk of disease progression compared to AC group (P=0.004), but there was no statistical significance in OS. Therefore, we further plotted the PFS and OS curves between groups in the PD- L1-negative subgroup (**Figures 9**, **10**).

### 3.5 Confirmed objective response

The response to treatment in the three groups is shown in **Table 4**, and no patient was able to achieve CR. The ORR in AC, PC and APC group were 22.81%, 30.43% and 24.32%, respectively (P = 0.595). The DCR in AC and PC group was not statistically significant (DCR: 82.46% vs 92.75%, P = 0.076). The patients in APC group had a better DCR than patients in AC group (DCR: 97.30% vs 82.46%, P = 0.045).

**Table 4:** Summary of confirmed response assessed by RECIST version 1.1.

Confirmed response	AC	PC	APC
Best response			
CR	0	0	0
PR	13 (22.81%)	21 (30.43%)	9 (24.32%)
SD	34 (59.65%)	43 (62.32%)	27 (72.98%)
PD	10 (17.54%)	5 (7.25%)	1 (2.70%)
ORR	22.81%	30.43%	24.32%
*P*	0.595		
DCR	82.46%	92.75%	97.30%
*P*	0.054		
PC *vs.* AC	0.076		
APC *vs.* AC	0.045*		

Symbol * means that variables are significant with p value < 0.05.

RECIST, Response Evaluation Criteria In Solid Tumors.

ORR, Objective response rate;CR, Complete response; PR, Partial response (PR).

DCR, Disease control rate; CR, Complete response; PR, Partial response; SD, Stable disease.

AC group, Anti-angiogenic agents plus chemotherapy.

PC group, PD-1/PD-L1 inhibitors plus chemotherapy.

APC group, Anti-angiogenic agents plus PD-1/PD-L1 inhibitors plus chemotherapy.

### 3.6 Toxicity

Adverse events in each treatment group are listed in **Tables 5**, **6**. Hematologic toxicity was the most common adverse reaction which mainly resulted from chemotherapy, resulting in no difference between the groups. Most of the adverse reactions were grade 1-2, with relatively few grade 3–4 adverse reactions. Of the grade 1–2 adverse reactions, a total of 19 patients (20.2%) in AC and APC group developed proteinuria and 11 patients (11.7%) developed treatment-related hypertension, considered as anti-angiogenic-specific adverse events. Rash occurred in 4 patients (3.8%), pneumonia in 10 patients (9.4%), hypothyroidism in 16 patients (15.1%) and immune myocarditis in 10 patients (9.4%) in PC and APC group, considering the unique adverse events of PD-1/PD-L1 inhibitors. Overall, the incidence of grade 1-2 adverse reactions was similar in AC and PC group (73.7% vs 75.4%, P=0.829), with a significantly higher incidence in APC group than in AC group (94.6% vs 73.7%, P=0.010). Grade 3–4 adverse reactions in AC (64.9%), PC (68.1%) and APC (70.3%) group had similar incidence rate. No patient died from AEs.

**Table 5:** Adverse events.

Events	Grade 1–2				Grade 3–4			
	AC	PC	APC	p value	AC	PC	APC	p value
Leukopenia	12 (21.1%)	12 (17.4%)	7 (18.9%)	0.873	8 (14.0%)	6 (8.7%)	2 (5.4%)	0.410
Pneumonia	0 (0%)	7 (10.1%)	3 (8.1%)	0.024*	0 (0%)	0 (0%)	0 (0%)	/
Transaminases increased	9 (15.8%)	17 (24.6%)	10 (27.0%)	0.350	0 (0%)	0 (0%)	0 (0%)	/
Hypothyroidism	0 (0%)	9 (13.0%)	7 (18.9%)	0.001*	0 (0%)	2 (2.9%)	0 (0%)	0.509
Rash	0 (0%)	2 (2.9%)	2 (5.4%)	0.212	0 (0%)	0 (0%)	0 (0%)	/
Vomiting	3 (5.3%)	3 (4.3%)	2 (5.4%)	1.000	0 (0%)	0 (0%)	0 (0%)	/
Thrombocytopenia	4 (7.0%)	2 (2.9%)	5 (13.5%)	0.091	3 (5.3%)	3 (4.3%)	2 (5.4%)	1.000
Hypopituitarism	0 (0%)	0 (0%)	0 (0%)	/	0 (0%)	0 (0%)	0 (0%)	/
Myocarditis	0 (0%)	8 (11.6%)	2 (5.4%)	0.016*	0 (0%)	0 (0%)	0 (0%)	/
Proteinuria	11 (19.3%)	0 (0%)	8 (21.6%)	<0.001*	0 (0%)	0 (0%)	0 (0%)	/
Constipation	3 (5.3%)	7 (10.1%)	6 (16.2%)	0.214	0 (0%)	0 (0%)	0 (0%)	/
Insomnia	1 (1.8%)	0 (0%)	1 (2.7%)	0.331	0 (0%)	0 (0%)	0 (0%)	/
Epistaxis	0 (0%)	0 (0%)	2 (5.4%)	0.050	0 (0%)	0 (0%)	0 (0%)	/
Hypertension	7 (12.3%)	0 (0%)	4 (10.8%)	0.003*	0 (0%)	0 (0%)	0 (0%)	/
Anemia	6 (10.5%)	6 (8.7%)	5 (13.5%)	0.718	4 (7.0%)	6 (8.7%)	5 (13.5%)	0.539
Thrombosis	0 (0%)	0 (0%)	0 (0%)	/	0 (0%)	0 (0%)	0 (0%)	/
Capillary proliferation	0 (0%)	0 (0%)	2 (5.4%)	0.050	0 (0%)	0 (0%)	0 (0%)	/
Fever	7 (12.3%)	6 (8.7%)	5 (13.5%)	0.656	0 (0%)	0 (0%)	0 (0%)	/
Neutropenia	6 (10.5%)	6 (8.7%)	4 (10.8%)	0.891	7 (12.3%)	8 (11.6%)	4 (10.8%)	1.000

Symbol * means that variables are significant with p value < 0.05.

**Table 6:** Different grades of adverse events.

AE	AC group	PC group	APC group
Grade 1-2	42 (73.7%)	52 (75.4%)	35 (94.6%)
PC vs AC	0.829		
APC vs AC	0.010		
P	0.031		
Grade 3-4	37 (64.9%)	47 (68.1%)	26 (70.3%)
PC vs AC	0.704		
APC vs AC	0.589		
P	0.854		

## 4 Discussion

Either Programmed cell death protein 1 (PD-1)/Programmed cell death-Ligand protein 1 (PD-L1) inhibitors plus chemotherapy or anti-angiogenic agents plus chemotherapy has become first-line therapy in negative or low PD-L1 patients with driver-gene-negative metastatic lung adenocarcinoma. PD-1/PD-L1 inhibitors plus anti-angiogenic agents with chemotherapy is also an option.

In this single-center retrospective study, we reported a total of 163 driver-negative advanced lung adenocarcinoma patients with PD-L1 TPS ≤49% who received first-line treatment. The results demonstrated that the PFS of PD-1/PD-L1 inhibitors plus anti-angiogenic agents with chemotherapy was superior to that of anti-angiogenic agents plus chemotherapy and PD-1/PD-L1 inhibitors plus chemotherapy. There was no significant difference in OS between the three groups from the perspective of long survival.

The clinical benefit of PD-1/PD-L1 inhibitors is currently evident in patients with high PD-L1 expression, but the therapeutic effect in patients with low or negative PD-L1 expression is still not explicit. Bevacizumab in combination with chemotherapy as first-line therapy is also recommended by guidelines. However, there is not enough clinical evidence to guide the choice of the optimal regimen in metastatic lung adenocarcinoma with PD-L1 TPS <50%. Mechanistically, the efficacy of PD-1/PD-L1 inhibitors is influenced by the level of PD-L1 expression, and the process of blocking the interaction of VEGF ligands with receptors by anti-angiogenic drugs is not influenced by the level of PD-L1 expression (12). Therefore, PD-1/PD-L1 inhibitors plus anti-angiogenic agents have the potential to be an effective option for patients with low or negative PD-L1 expression. Based on current clinical evidence, we collected three first-line treatment regimens in this population: anti-angiogenic agents plus chemotherapy (AC group), PD-1/PD-L1 inhibitors plus chemotherapy (PC group) and PD-1/PD-L1 inhibitors plus anti-angiogenic agents with chemotherapy (APC group), aiming at exploring rationalized treatment modalities through head-to-head comparison.

Global Multicenter Express Study demonstrated that the percentage of PD-L1 TPS ≥ 50% and TPS ≥ 1% were respectively 27% and 53% in 1064 Patients with NSCLC whose EGFR Mutations and ALK Alterations were negative (13). Express II Study explored PD-L1 expression in Chinese patients with stage IIIB/IV NSCLC and found that 48.2% had PD-L1 TPS <1% and >70% were driver-gene negative (14). Median PFS of patients with EGFR wild-type advanced non-squamous NSCLC treated with first-line bevacizumab plus platinum-based dual-agent chemotherapy was 8.3 months in the BEYOND study (15), which was similar to our study (mPFS=7.0months). The Camel, ORIENT-11, Impower 130 and KEYNOTE 189 trials revealed superior benefits of PD-1/PD-L1 inhibitors plus chemotherapy over chemotherapy alone, regardless of PD-L1 expression (16–19). The IMPOWER 150 trial demonstrated improved PFS for atezolizumab plus bevacizumab with chemotherapy versus bevacizumab plus chemotherapy (8.3 months vs. 6.8 months, HR: 0.62, P<0.001) (9, 20). Our results found that the median PFS was better in APC group than in AC group (13.8 months vs 6.3 month) and PC group (13.8 months vs 8.7 months). Meanwhile, our further analysis in the PD-L1-negative subgroup revealed that the four-drug combination regimen of immune checkpoint inhibitors, bevacizumab and chemotherapy had a better PFS than the other two regimens, but no difference was observed in the PD-L1 TPS 1-49%, which may be affected by the sample size.

The IMPOWER 150 trial is the first study to compare the efficacy of two regimens, PD-1/PD-L1 inhibitors plus chemotherapy and anti-angiogenic agents plus chemotherapy, in patients with low PD-L1 expression. However, there was no significant difference of OS in the PD-L1-negative subgroup analysis using the two regimes. Our study also obtained similar conclusions, which showed no significant difference in survival between first-line use of PD-1/PD-L1 inhibitors and anti-angiogenic drugs in patients with metastatic lung adenocarcinoma with PD-L1 TPS <50%. All these provided evidence for the use of anti-angiogenic drugs plus chemotherapy in patients with contraindications to PD-1/PD-L1 inhibitors, and similarly, the use of PD-1/PD-L1 inhibitors is not a bad option for patients with diseases such as severe hypertension and renal dysfunction.

The results of this study showed no significant difference in survival between first-line use of immunocombination chemotherapy and combination chemotherapy with antiangiogenic agents in advanced lung adenocarcinoma patients with PD-L1 TPS ≤49%, which provides evidence for the use of antiangiogenic agents in patients with contraindications to PD-1/PD-L1 inhibitors, and similarly, in patients with severe hypertension and renal dysfunction and other disorders that make them inappropriate candidates for the use of antiangiogenic agents, immunotherapy is also an option. In terms of safety, most patients had an adverse reaction grade of 1-2, and although the incidence of grade 1–2 adverse reactions was significantly higher in the four-drug combination regimen (APC group) than in the AC and PC groups, there was no significant difference between the APC group and the other two groups in terms of grade 3–4 serious adverse reactions. In this study, immune-related adverse reactions were mainly rash, pneumonia, hypothyroidism and myocarditis, and anti-angiogenic drug adverse reactions were mainly treatment-related hypertension and proteinuria. In short-term efficacy, the APC group had a certain advantage, whereas the short-term efficacy of the AC group and the PC group was comparable, but there was no significant difference in OS between the three groups. Combined with the adverse effects, patients with better physical ability may be preferred to use the four-drug combination, while when the effects of adverse effects on patients need to be considered, chemotherapy combined with immune checkpoint inhibitors or anti-angiogenic drugs may be selected according to the patient’s condition, and this study provides safety support for the use of alternative regimens for patients with contraindications to the corresponding drug therapy.

In our study, multifactorial cox regression analysis showed that ECOG score, smoking history, and liver metastasis were independent prognostic factors for PFS and gender, ECOG score and bone metastasis were independent prognostic factors for OS. Smoking is significantly associated with decreased survival in lung cancer, and several studies have confirmed this finding (21). The majority of NSCLC patients currently undergoing immunotherapy are in good physical status, and there is still no optimal regimen for patients in poor physical status (22). Early on, it was demonstrated that patients with an initial physical status of 0, no bone metastases, female, and no liver metastases were predictors of longer survival (23). In this study, patients with ECOG score of 0–1 had significantly better prognosis than those with ECOG score of 2. Patients with liver metastases have the worst prognosis in metastatic lung cancer, and liver metastases occur in about 15% of patients with poor prognosis of metastatic NSCLC, which may be related to the poor response to chemotherapy in patients with liver metastases, among other reasons (24). Meanwhile, patients with bone metastases have poorer survival, which is mostly thought to be related to low survival due to skeletal-related events such as pathological fractures, spinal cord compression and hypercalcaemia in malignancy (25, 26), and the improved survival of patients with bone metastases has been attributed to the clinical use of zoledronic acid (27). Although Cox analysis showed that adrenal metastasis was associated with poor prognosis, it was not an independent prognostic factor, and some studies have shown that adrenal metastasis is an unfavorable factor, the reason for this is uncertain, and further studies are needed to explore the impact of adrenal metastasis on survival. Metastases in different anatomical locations may be associated with different clinical outcomes and treatment responses in NSCLC, so it is clinically important to develop a more individualized treatment plan that takes into account the patient’s organ metastases. However, in this study, the differences between patients’ treatment regimens, the inconsistent follow-up time of different regimens, and the inherent flaws of retrospective nature all have an impact on the results of Cox analysis, so subsequent subdivision of treatment regimens is needed to explore the impact of therapeutic agents on the survival of NSCLC with different organ metastases.

This study optimized the first-line treatment regimen for patients with advanced lung adenocarcinoma who were negative for driver genes and had low or negative PD-L1 expression, but as a retrospective study, this topic still has several shortcomings. Firstly, the specimens sent for examination included both primary and metastatic specimens, and the detected PD-L1 levels were not completely standardized. Secondly, because this study was a retrospective study with a limited sample size collected, the results of the study may be biased from the real situation. Additionally, due to the insufficient follow-up time, the median OS of the patients in the combination of immune checkpoint inhibitors, anti-angiogenesis and chemotherapy group was not reached, which had an impact on the study completeness. Furthermore, immunological and chemotherapeutic agents were not aligned, which may have affected survival and thus biased the study results. Therefore, more rigorous large prospective studies are needed to further explore this in the future.

## 5 Conclusion

Among the first-line treatment options for patients with advanced lung adenocarcinoma who are driver-negative and have negative or low expression of PD-L1: 1. There is no significant difference in the survival efficacy between PD-1/PD-L1 inhibitors plus chemotherapy and anti-angiogenic agents plus chemotherapy. Therefore, anti-angiogenic agents plus chemotherapy can be chosen for patients with contraindications to PD-1/PD-L1 inhibitors, and for patients with diseases such as severe hypertension and renal abnormalities, PD-1/PD-L1 inhibitors plus chemotherapy can be a choice. 2. PD-1/PD-L1 inhibitors plus anti-angiogenic agents with chemotherapy regimens can provide better PFS compared with the other two regimens. Therefore, it is recommended to use this regimen as the first-line treatment for patients who are able to tolerate it. 3. There is no significant difference in long-term survival among the three regimens, and the safety of the three regimens is controllable, so the decision to use them can be made individually according to the contraindications of the patients.

## Data Availability

The raw data supporting the conclusions of this article will be made available by the authors, without undue reservation.

## Supplementary material

Supplementary Figure 1 Effect of different clinical characteristics on patients’ disease progression. **(A)** ECOG core **(B)** Smoking history **(C)** Liver metastasis **(D)** Bone metastasis.

Supplementary Figure 2 Effect of different clinical characteristics on patients’ survival. **(A)** ECOG score **(B)** Gender **(C)** Bone metastasis **(D)** Adrenal metastasis.

## References

[1] Snee M Cheeseman S Thompson M Riaz M Sopwith W Lacoin L . Treatment patterns and survival outcomes for patients with non-small cell lung cancer in the UK in the preimmunology era: a REAL-Oncology database analysis from the I-O Optimise initiative. BMJ Open. (2021) 11:e046396. doi: 10.1136/bmjopen-2020-046396. PMID: 34526333 PMC8444261

[2] Ramalingam SS Vansteenkiste J Planchard D Cho BC Gray JE Ohe Y . Overall survival with osimertinib in untreated, EGFR-mutated advanced NSCLC. N Engl J Med. (2020) 382:41–50. doi: 10.1056/nejmoa1913662. PMID: 31751012

[3] Shaw AT Bauer TM de Marinis F Felip E Goto Y Liu G . First-line lorlatinib or crizotinib in advanced ALK-positive lung cancer. N Engl J Med. (2020) 383:2018–29. doi: 10.1016/j.lungcan.2022.11.004. PMID: 33207094

[4] Reck M Rodríguez-Abreu D Robinson AG Hui R Csőszi T Fülöp A . Five-year outcomes with pembrolizumab versus chemotherapy for metastatic non-small-cell lung cancer with PD-L1 tumor proportion score ≥ 50. J Clin Oncol. (2021) 39:2339–49. doi: 10.1200/jco.21.00174. PMID: 33872070 PMC8280089

[5] Reck M Rodríguez-Abreu D Robinson AG Hui R Csőszi T Fülöp A . Pembrolizumab versus chemotherapy for PD-L1-positive non-small-cell lung cancer. N Engl J Med. (2016) 375:1823–33. doi: 10.1056/nejmoa1606774. PMID: 27718847

[6] Mok TSK Wu YL Kudaba I Kowalski DM Cho BC Turna HZ . Pembrolizumab versus chemotherapy for previously untreated, PD-L1-expressing, locally advanced or metastatic non-small-cell lung cancer (KEYNOTE-042): a randomised, open-label, controlled, phase 3 trial. Lancet. (2019) 393:1819–30. doi: 10.1016/s0140-6736(18)32409-7. PMID: 30955977

[7] Paz-Ares L Vicente D Tafreshi A Robinson A Soto Parra H Mazières J . A randomized, placebo-controlled trial of pembrolizumab plus chemotherapy in patients with metastatic squamous NSCLC: Protocol-specified final analysis of KEYNOTE-407. J Thorac Oncol. (2020) 15:1657–69. doi: 10.1016/j.jtho.2020.06.015. PMID: 32599071

[8] Herbst RS Giaccone G de Marinis F Reinmuth N Vergnenegre A Barrios CH . Atezolizumab for first-line treatment of PD-L1-selected patients with NSCLC. N Engl J Med. (2020) 383:1328–39. doi: 10.1056/nejmoa1917346. PMID: 32997907

[9] Socinski MA Nishio M Jotte RM Cappuzzo F Orlandi F Stroyakovskiy D . IMpower150 final overall survival analyses for atezolizumab plus bevacizumab and chemotherapy in first-line metastatic nonsquamous NSCLC. J Thorac Oncol. (2021) 16:1909–24. doi: 10.1016/j.jtho.2021.07.009. PMID: 34311108

[10] Tyagi P . Bevacizumab, when added to paclitaxel/carboplatin, prolongs survival in previously untreated patients with advanced non-small-cell lung cancer: preliminary results from the ECOG 4599 trial. Clin Lung Cancer. (2005) 6:276–8. doi: 10.1016/s1525-7304(11)70220-0. PMID: 15845177

[11] Sandler A Gray R Perry MC Brahmer J Schiller JH Dowlati A . Paclitaxel-carboplatin alone or with bevacizumab for non-small-cell lung cancer. N Engl J Med. (2006) 355:2542–50. doi: 10.1056/nejmoa061884. PMID: 17167137

[12] Tian L Goldstein A Wang H Ching Lo H Sun Kim I Welte T . Mutual regulation of tumour vessel normalization and immunostimulatory reprogramming. Nature. (2017) 544:250–4. doi: 10.1038/nature21724. PMID: 28371798 PMC5788037

[13] Dietel M Savelov N Salanova R Micke P Bigras G Hida T . Real-world prevalence of programmed death ligand 1 expression in locally advanced or metastatic non-small-cell lung cancer: The global, multicenter EXPRESS study. Lung Cancer. (2019) 134:174–9. doi: 10.1016/j.lungcan.2019.06.012. PMID: 31319978

[14] Lin D Yang X Jiang L Wang W Hou Y Li Y . P33.12 real-world prevalence of PD-L1 expression in Chinese patients with advanced or metastatic NSCLC: Express II study. J Thorac Oncol. (2021) 16:S410. doi: 10.1016/j.jtho.2021.01.681. PMID: 38826717

[15] Zhou C Wu YL Chen G Liu X Zhu Y Lu S . BEYOND: a randomized, double-blind, placebo-controlled, multicenter, phase III study of first-line carboplatin/paclitaxel plus bevacizumab or placebo in Chinese patients with advanced or recurrent nonsquamous non-small-cell lung cancer. J Clin Oncol. (2015) 33:2197–204. doi: 10.1200/jco.2014.59.4424. PMID: 26014294

[16] Zhou C Chen G Huang Y Zhou J Lin L Feng J . Camrelizumab plus carboplatin and pemetrexed versus chemotherapy alone in chemotherapy-naive patients with advanced non-squamous non-small-cell lung cancer (CameL): a randomised, open-label, multicentre, phase 3 trial. Lancet Respir Med. (2021) 9:305–14. doi: 10.1016/s2213-2600(20)30365-9. PMID: 33347829

[17] Lu S Wang J Yu Y Yu X Hu Y Ai X . Tislelizumab plus chemotherapy as first-line treatment for locally advanced or metastatic nonsquamous NSCLC (RATIONALE 304): a randomized phase 3 trial. J Thorac Oncol. (2021) 16:1512–22. doi: 10.1016/j.jtho.2021.05.005. PMID: 34033975

[18] West H McCleod M Hussein M Morabito A Rittmeyer A Conter HJ . Atezolizumab in combination with carboplatin plus nab-paclitaxel chemotherapy compared with chemotherapy alone as first-line treatment for metastatic non-squamous non-small-cell lung cancer (IMpower130): a multicentre, randomised, open-label, phase 3 trial. Lancet Oncol. (2019) 20:924–37. doi: 10.1016/s1470-2045(19)30167-6. PMID: 31122901

[19] Gadgeel S Rodríguez-Abreu D Speranza G Esteban E Felip E Dómine M . Updated analysis from KEYNOTE-189: Pembrolizumab or placebo plus pemetrexed and platinum for previously untreated metastatic nonsquamous non-small-cell lung cancer. J Clin Oncol. (2020) 38:1505–17. doi: 10.1200/jco.19.03136. PMID: 32150489

[20] Socinski MA Jotte RM Cappuzzo F Orlandi F Stroyakovskiy D Nogami N . Atezolizumab for first-line treatment of metastatic nonsquamous NSCLC. N Engl J Med. (2018) 378:2288–301. doi: 10.1056/nejmoa1716948. PMID: 29863955

[21] Pinsky PF Kramer BS . Lung cancer risk and demographic characteristics of current 20–29 pack-year smokers: Implications for screening. J Natl Cancer Inst. (2015) 107. doi: 10.1093/jnci/djv226. PMID: 26483244 PMC4849359

[22] Facchinetti F Di Maio M Perrone F Tiseo M . First-line immunotherapy in non-small cell lung cancer patients with poor performance status: a systematic review and meta-analysis. Transl Lung Cancer Res. (2021) 10:2917–36. doi: 10.21037/tlcr-21-15. PMID: 34295688 PMC8264315

[23] Finkelstein DM Ettinger DS Ruckdeschel JC . Long-term survivors in metastatic non-small-cell lung cancer: an Eastern Cooperative Oncology Group study. J Clin Oncol. (1986) 4:702–9. doi: 10.1200/jco.1986.4.5.702. PMID: 3701389

[24] Görg C Schwerk WB Wolf M Havemann K . Prognostic value of response to chemotherapy using ultrasound in lung cancer with metastatic liver involvement. Bildgebung. (1990) 57:70–3. 1965423

[25] Riihimäki M Hemminki A Fallah M Thomsen H Sundquist K Sundquist J . Metastatic sites and survival in lung cancer. Lung Cancer. (2014) 86:78–84. doi: 10.1016/j.lungcan.2014.07.020 25130083

[26] Saad F Lipton A Cook R Chen YM Smith M Coleman R . Pathologic fractures correlate with reduced survival in patients with Malignant bone disease. Cancer. (2007) 110:1860–7. doi: 10.1002/cncr.22991. PMID: 17763372

[27] Zarogoulidis K Boutsikou E Zarogoulidis P Eleftheriadou E Kontakiotis T Lithoxopoulou H . The impact of zoledronic acid therapy in survival of lung cancer patients with bone metastasis. Int J Cancer. (2009) 125:1705–9. doi: 10.1002/ijc.24470. PMID: 19521984

