# Supplementary figures and images for: Clinical exploration of first-line therapy in metastatic lung adenocarcinoma patients with negative or low PD-L1 expression: a retrospective cohort study
Source: Front Immunol. 2026 Jun 3;17:1836760. doi: 10.3389/fimmu.2026.1836760 (PMC13272393; doi:10.3389/fimmu.2026.1836760)

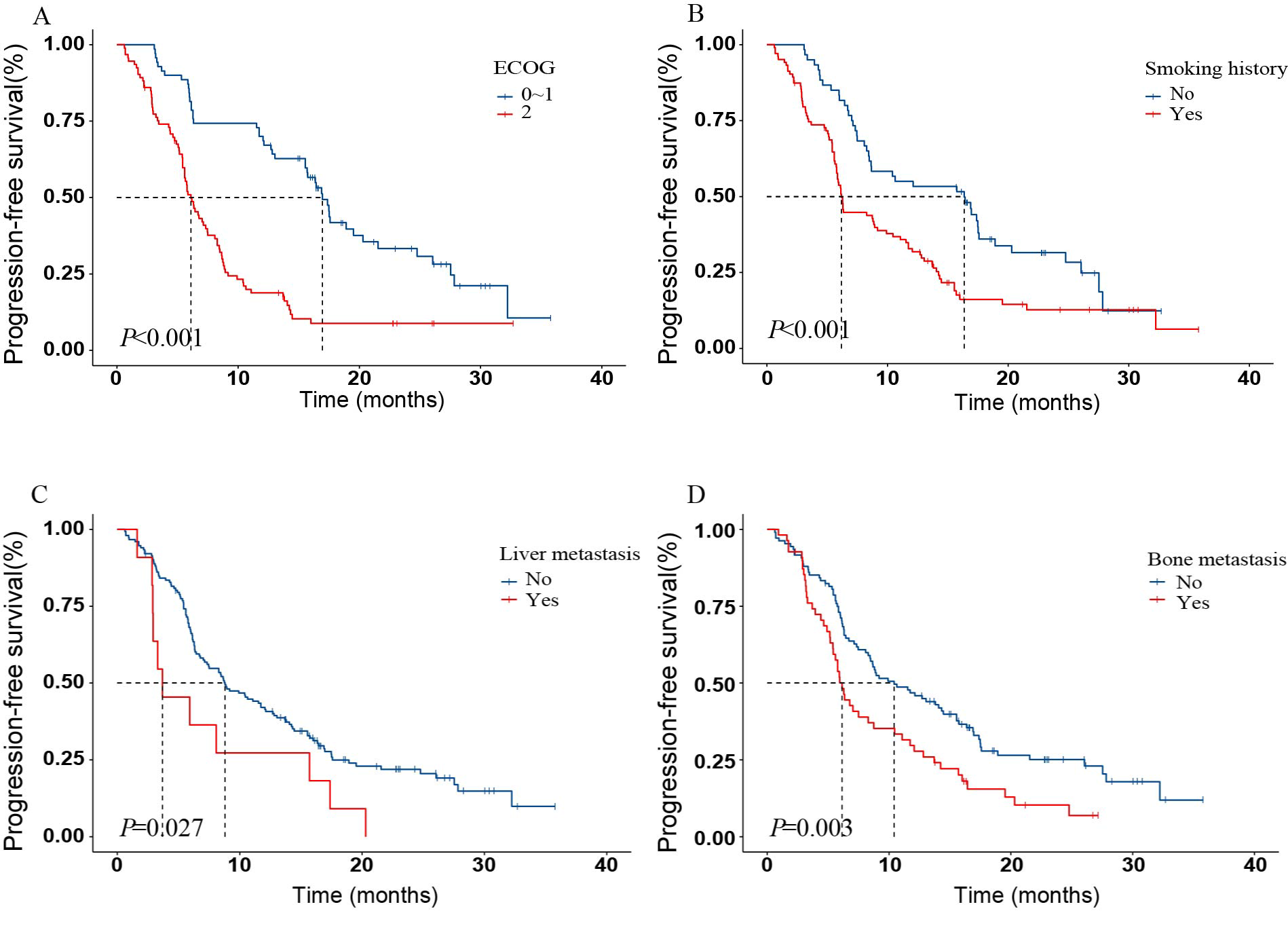

Supplement: Supplementary Figure 1 — Effect of different clinical characteristics on patients’ disease progression. (A) ECOG core (B) Smoking history (C) Liver metastasis (D) Bone metastasis. [file Image1.tif]

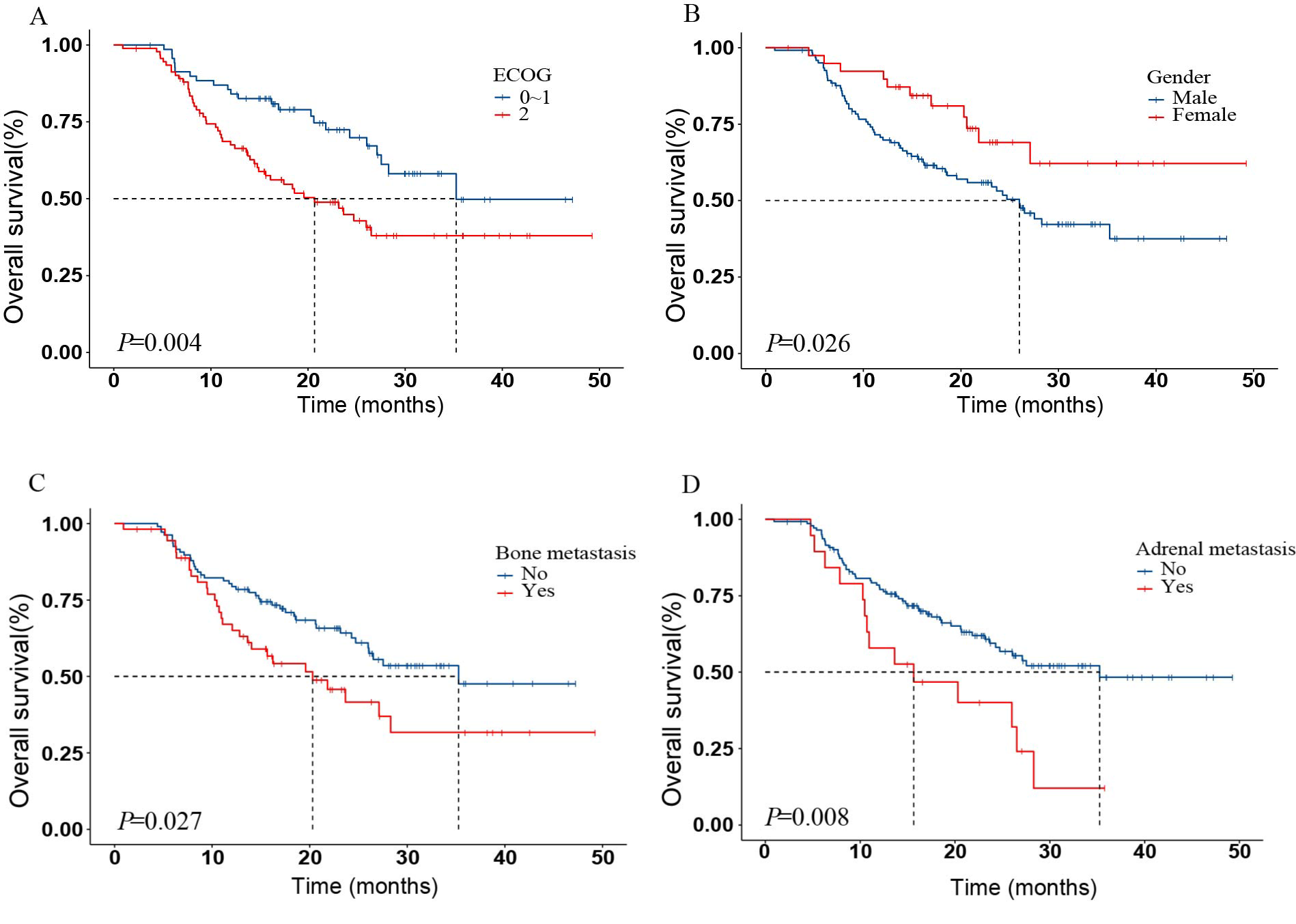

Supplement: Supplementary Figure 2 — Effect of different clinical characteristics on patients’ survival. (A) ECOG score (B) Gender (C) Bone metastasis (D) Adrenal metastasis. [file Image2.tif]
